# Kaposi's Sarcoma-Associated Herpesvirus-Related Solid Lymphoma Involving the Heart and Brain

**DOI:** 10.1155/2011/729854

**Published:** 2011-04-03

**Authors:** Jason R. Andrews, Yoon Andrew Cho-Park, Judith Ferry, Jeremy S. Abramson, Gregory K. Robbins

**Affiliations:** ^1^Division of Infectious Diseases, Massachusetts General Hospital, 55 Fruit Street, Cox 506, Boston, MA 02114, USA; ^2^Harvard Medical School, Boston, MA 02115, USA; ^3^Department of Neurology, Massachusetts General Hospital, Boston, MA 02114, USA; ^4^Department of Neurology, Brigham and Women's Hospital, Boston, MA 02115, USA; ^5^Department of Pathology, Massachusetts General Hospital, Boston, MA 02114, USA; ^6^Cancer Center, Massachusetts General Hospital, Boston, MA 02114, USA

## Abstract

Since its discovery in 1994, Kaposi's sarcoma-associated herpesvirus (KSHV) has been associated with lymphoproliferative disorders, particularly in patients infected with human immunodeficiency virus (HIV). The disorders most strongly linked to KSHV are multicentric Castleman's Disease (MCD), primary effusion lymphoma, and diffuse large B-cell lymphomas. We report an unusual case of KSHV-associated lymphoma in an HIV-infected patient manifesting with myocardial and central nervous system involvement. We discuss this case in the context of increasing array of KSHV-associated lymphomas. In the HIV-infected patient with a mass lesion, a history of cutaneous Kaposi's sarcoma and prolonged immunosuppression should alert clinicians as to the possibility of KSHV-associated lymphoproliferative disorders, in order to establish a timely diagnosis.

## 1. Case Report

A 46-year-old man with HIV and a history of cutaneous Kaposi's sarcoma presented with left eyelid droop and left-sided facial numbness five days after restarting antiretroviral therapy following a long hiatus. He was diagnosed with HIV 21 years prior and had a nadir CD4 cell count of 107. His CD4 cell count when he restarted his antiretroviral regimen was 159 cells/mm^3^, viral load 511,000 c/mL. He had been intermittently compliant with antiretroviral therapy in the past and since resumption of therapy. In 2005, he had a recurrence of Kaposi's sarcoma and received five cycles of paclitaxel with clinical remission by 2006. Physical examination was otherwise unremarkable at the time and magnetic resonance imaging (MRI) of the brain was read as having no significant abnormalities. His symptoms initially were stable, but in the subsequent two months, they worsened, with hypoesthesia and paresthesia over all three branches of the trigeminal nerve on the left, severe headaches, ataxia, dysarthria, and dysphagia. He also began to experience fatigue, weight loss, and night sweats. A repeat MRI revealed enhancing leptomeningeal masses with associated mass effect involving the trigeminal nerve, middle cerebellar peduncle, pons, and upper spinal cord. Review of his prior MRI showed a suggestion of enhancement of the left trigeminal nerve intradurally. 

A lumbar puncture revealed elevated protein (205 g/dL), slightly depressed glucose (45 g/dL), with 106 leukocytes (32% lymphocytes, 26% monocytes, and 42% unclassified cells), and no erythrocytes. Cerebral spinal fluid (CSF) cytology was notable for atypical appearing cells, which were suspicious for, but not sufficient for a primary diagnosis of malignancy (see Figure 2(a)). CSF flow cytometry demonstrated no clonal B- or T-cell populations, and *IGH* gene rearrangement was not detected by PCR. CSF PCR for EBV was negative.

**Figure 1:**
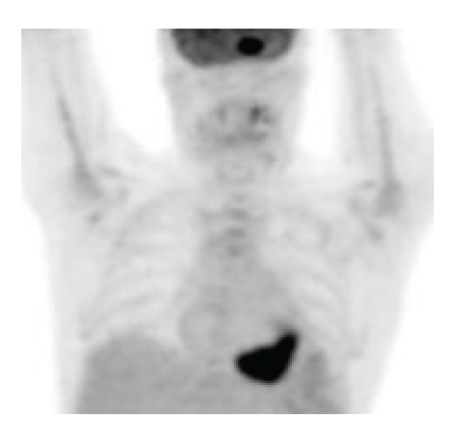
PET-CT demonstrated a focus of avidity at the known cerebellopontine lesion, as well as in the posterior inferior wall and papillary muscle of the left ventricle of the heart.

**Figure 2:**
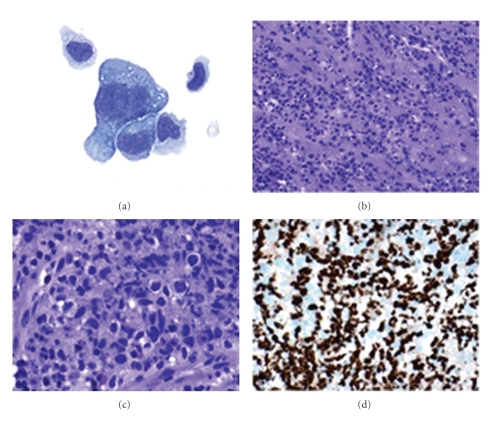
(a) Cerebrospinal fluid contains rare large, atypical cells with lobated nuclei and abundant basophilic cytoplasm; scattered smaller nonneoplastic cells are also present. (b)–(d) Endomyocardial biopsy. Microscopic examination by hematoxylin and eosin stain shows an atypical lymphoid proliferation infiltrating the myocardium (b). Higher power revealed large atypical discohesive cells with few admixed eosinophils (c). Large lymphoid cells stained avidly for Kaposi's sarcoma herpesvirus by immunostain (d).

A bone marrow biopsy was unremarkable and core needle biopsy of a modestly enlarged retroperitoneal lymph node showed no evidence of lymphoproliferative disease. There was no evidence of pleural, peritoneal, or pericardial effusions on computed tomography and ultrasound studies. A positron emission tomography with computed tomography (PET/CT) was then performed and demonstrated increased uptake at the known cerebellopontine lesion, as well as in the posterior inferior wall and papillary muscle of the left ventricle of the heart (Figure 1). An endomyocardial biopsy of the left ventricle was performed via left heart catheterization. Pathology revealed large atypical discohesive cells infiltrating the myocardium. Immunostains of the atypical lymphoid cells were strongly KSHV+ (Figure 2), MUM1/IRF4+, CD79a rare+, CD20−. In situ hybridization for Epstein-Barr virus (EBV) using a probe for EBER showed no staining. Comparison with the CSF specimens showed that these atypical cells were morphologically similar to the suspicious cells in the CSF (Figure 2(a)).

A preliminary diagnosis of large B-cell lymphoma was made based on morphologic appearance, and the patient was initiated on treatment with R-CHOP (rituximab, cyclophosphamide, doxorubicin, vincristine, and prednisone) along with intrathecal methotrexate, cytarabine, and hydrocortisone for the leptomeningeal disease. He was observed on telemetry for one week without cardiac ectopy and was discharged home. One day following discharge, he had syncope and was found to be in ventricular tachycardia with preserved blood pressure, for which he was treated with lidocaine and amiodarone with successful conversion to sinus rhythm. CD20 staining on his pathology returned negative and so treatment with cycle 2 was changed to EPOCH (etoposide, prednisone, vincristine, cyclophosphamide, and doxorubicin) without rituximab. High-dose systemic methotrexate was included to treat the CNS disease. Six weeks into treatment, restaging PET CT imaging showed no persistent FDG-avid disease of the heart, and MRI showing significant resolution of his intracranial tumor. Spinal fluid examination demonstrated no persistent circulating disease in the CSF. Clinically, his headaches, dysarthria, and dysphagia improved, but he still had numbness of his left face along the V1 and V2 distribution of the trigeminal nerve along with an upper motor facial nerve palsy on the left. Some residual gait ataxia was notable on examination.

## 2. Discussion

Kaposi's sarcoma was first described in elderly European men in 1872 by Moritz Kaposi, and lymphomatous effusions were identified in patients with HIV in the late 1980s [1], but the common viral etiology was not identified until 1994 [2]. Since that time, an array of lymphoproliferative manifestations of KSHV infection have been recognized, with most cases being identified in patients with HIV infection. The most well-characterized diseases are multicentric Castleman's disease (MCD) [3, 4], diffuse large B-cell lymphomas arising from MCD, and primary effusion lymphoma (PEL), for which KSHV detection is the *sine qua non* [5]. More recently, cases of “solid” or “extracavitary” lymphomas with PEL-like pathologic features have been reported [6]. There is conflicting evidence as to whether these tumors carry a worse prognosis than PEL. Chadburn et al. described eight patients with KSHV-solid lymphomas that were indistinguishable on immunophenotype and genotype from PEL, with a median survival of 11 months compared with 3 months for PEL [6]. Simonelli et al. described three patients with solid-variant PEL, all of whom died within a month of diagnosis (median survival of 22 days) [7]. Successful treatment with antiretroviral therapy and chemotherapy has been reported [6, 8].

The patient we report here had a large B-cell lymphoma with features of extracavitary PEL. In the initial description of PEL, all tumors stained positive for Epstein-Barr virus (EBV) in addition to KSHV, and subsequent case reports have confirmed EBV coexpression in the majority of cases [6, 9, 10]. Our patient's tumor did not, consistent with prior reports suggesting that lack of EBV expression may occasionally be seen in extracavitary PEL [11].

KSHV-positive lymphomas have been recovered from a variety of solid organs, including the heart, bowel, lymph nodes, spermatic cord, skin, soft tissues, and spleen [12–14]. CNS involvement as is present in this case has rarely been reported in PEL, though it is common in other HIV-associated lymphomas. In addition, although immunosuppression is well recognized as a risk factor in the development of certain cardiac lymphomas, to our knowledge KSHV+ lymphoma involving the myocardium has not been described previously. This case highlights the importance of immunohistochemistry, HHV8, and EBV testing in the diagnosis of HIV-associated lymphomas, as morphologically this disease would otherwise be classified as diffuse large B-cell lymphoma, not otherwise specified.

The potential utility of antivirals in treatment of KSHV-associated lymphoproliferative disorders is an area of active interest. Ganciclovir has been shown to inhibit KSHV replication, and there have been case reports of its successful used alone or in adjunct to chemotherapy for MCD and PEL [15]. Oral valganciclovir may have a role in secondary prophylaxis for patients with PEL and other KSHV-associated lymphoproliferative disorders. 

Many patients presenting with KSHV-associated lymphomas have a history of or concurrent Kaposi's sarcoma [6, 11]. Our patient had two episodes of cutaneous Kaposi's sarcoma prior to his presentation with lymphoma. A history of Kaposi's sarcoma may be an important clue in the workup of B-symptoms and tumors identified in solid organs of patients with HIV. While KSHV viral load has been shown to have prognostic value in patient's with known Kaposi's sarcoma, there is limited data on the diagnostic or prognostic utility of KSHV viral loads in patients with suspected or known non-Hodgkin's lymphoma [7]. A small study of HIV patients with KSHV-associated lymphoproliferative disorders found that those with lymphoma had higher KSHV viral loads than those with MCD [7]. Use of antiretroviral therapy with viral load suppression, and higher CD4 cell counts have been shown to be protective from Kaposi's sarcoma and non-Hodgkin's lymphoma [16]. This case nicely highlights that any new mass lesion in a patient with a prior HHV8+ disease (KS, PEL, or MCD) warrants repeat tissue sampling given the propensity of multiple histologies to occur in the same patient, either concurrently or sequentially. 

In summary, this case illustrates an unusual presentation of a KSHV-associated lymphoma, presenting with central nervous system involvement, with diagnosis established on an endomyocardial biopsy, in contrast to the more common KSHV-associated lymphoproliferative disorders: MCD and PEL. We believe that intermittent nonadherence and prolonged episodes of immunosuppression may have put him at risk for KSHV-associated malignant transformation. Providers should be vigilant for atypical presentations of common malignancies in patients with prolonged or intermittent immunosuppression.
